# A case report of *Saksenaea vasiformis* mucormycosis infection of a lower segment caesarean section wound

**DOI:** 10.1016/j.ijscr.2025.111923

**Published:** 2025-09-08

**Authors:** Steven Nguyen, Hao Han Tan, Damian Fry

**Affiliations:** a Department of Surgery, Toowoomba Hospital, Toowoomba City, QLD, Australia

**Keywords:** Mucormycosis, Debridement, Fungal infection, Mesh repair, Dermal matrix

## Abstract

**Introduction:**

Mucormycosis is a rare and difficult condition to diagnose, often requiring histological confirmation. Only two previous case reports of mucormycosis infections following caesarean section have been published to date.

**Case presentation:**

A 24-year-old female from Australia presented with fevers, pain and discharge from her wound site seven days following a lower segment caesarean section. The patient failed to improve with broad-spectrum antibiotics and required radical surgical debridement. Tissue samples from the first debridement operation found necrotic fibroadipose tissue with fungal hyphae histologically. The hyphae were 90-degree branching with focal angioinvasion, a highly suggestive feature of mucormycosis, which eventually identified *Saksenaea vasiformis*.

**Discussion:**

The mucormycosis infection was treated with amphotericin B and posaconazole as well as multiple surgical debridement operations. Following resolution of the infection, reconstruction was performed with Phasix™ mesh repair of the abdominal fascia, in addition to biodegradable temporizing matrix (BTM) and split-thickness skin grafting.

**Conclusion:**

This case highlights the exceptionally rare diagnosis of mucormycosis in a caesarean section wound, especially in a developed country, and the complex multidisciplinary management required. Antifungal treatment and aggressive radical debridement were essential for treatment, as well as reconstruction in an infected setting.

## Highlights

• Mucormycosis is a rare and difficult condition to diagnose, which in this case report required histological analysis
• Treatment in this case involved a combination of liposomal amphotericin B and posaconazole
• Radical debridement of the lower segment caesarean section wound was required for the treatment of mucormycosis
• Abdominal wall reconstruction utilised Phasix mesh, biodegradable temporizing matrix, and split-thickness skin grafting

## 1 Introduction

Mucormycosis, also known as “black fungus”, is a rare and aggressive fungal infection that predominantly affects immunocompromised patients, such as those with poorly controlled diabetes mellitus. Mucormycosis refers to infections caused by members of the order of Mucorales, including *Rhizopus*, *Mucor*, and *Saksenaea* [1]. Typically, Mucorales represent a permanent part of the human environment, existing on wet organic material, as both spoiling and rotting agents, but also as material for cheeses and soy products [2]. Members of the order of Mucorales can cause cutaneous, pulmonary, rhino-orbital-cerebral or disseminated disease, all associated with high rates of mortality [3,4]. Unfortunately, mucormycosis remains a difficult and rare condition to diagnose, with only two case reports of mucormycosis infections following caesarean section published to date [5,6], with none in Australia.

This case report has been reported in line with the SCARE Criteria [7].

## 2 Case presentation

A 24-year-old female presented to a rural hospital emergency department seven days after an emergency caesarean section in rural Australia with acute worsening pain, erythema, and discharge from her incisional wound. The patient was septic on arrival, which required the initiation of broad-spectrum antibiotic treatment. The patient had no other significant past medical history, such as immunosuppression or diabetes mellitus. The indication for the patient's emergency caesarean section was for obstructed labour. This was complicated by primary postpartum haemorrhage (with an estimated blood loss of 1.8 L) from inadequate uterine tone, managed with a Bakri Balloon as well as prostaglandin and oxytocin infusions. After four days, she was transferred to our regional hospital under an obstetric team for persistent sepsis.

**Fig. 1:**
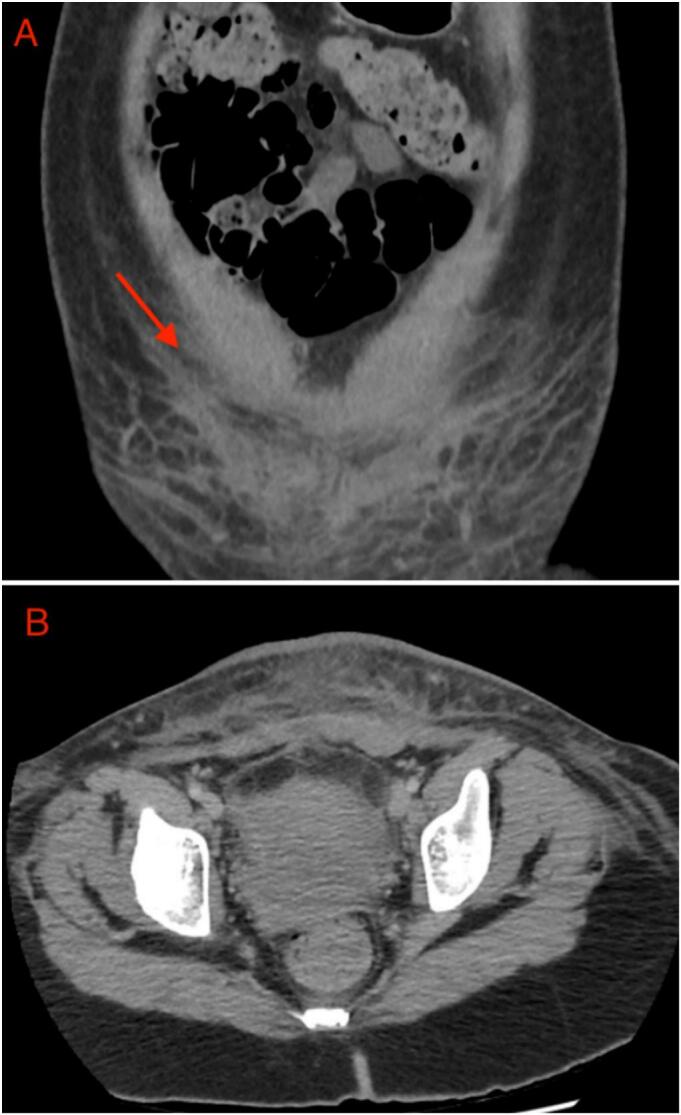
A-B. Coronal (A) and Axial (B) Computerised Tomography Imaging of Infected Pfannenstiel Wound with Marked Fat Stranding on admission to our regional centre.

Computerised tomography (CT) imaging of the abdomen and pelvis showed large amounts of fat stranding and scattered fluid around the lower abdomen with no intra-abdominal collections, as well as overlying skin thickening and bilateral inguinal lymphadenopathy (Fig. 1). The patient's white cell count was 9.7 × 10^9^/L and C-reactive protein was 330 mg/L on arrival to our regional hospital. Swabs of the infected site returned with scant anaerobic bacteria and mixed skin flora. Seven sets of blood cultures taken over the course of the patient's first six days from admission all returned negative for any microbial growth.

The patient continued to deteriorate despite broadening of antibiotic coverage to piperacillin-tazobactam, as well as vancomycin (which was later ceased due to an allergic reaction). Two days into admission at our regional hospital, a consult was sought from the general surgery team due to clinical evidence of severe soft tissue infection and inadequate response to antibiotic treatment. A decision was made for a surgical debridement of the wound. Intraoperative findings were a grossly infected incision with extensive soft tissue necrosis, concerning for necrotising fasciitis. However, no extension of infection into the rectus sheath or abdominal cavity was identified. Tissue samples and swabs from the wound were sent for microscopy, culture and histology, and an empirical necrotising fasciitis antibiotic regimen was initiated with the addition of Lincomycin. The wound was packed with gauze and dressed. Relook on post-operative day (POD) one showed a healthy wound and intact rectus sheath, and delayed primary closure was performed with drains on suction left in situ.

**Fig. 2:**
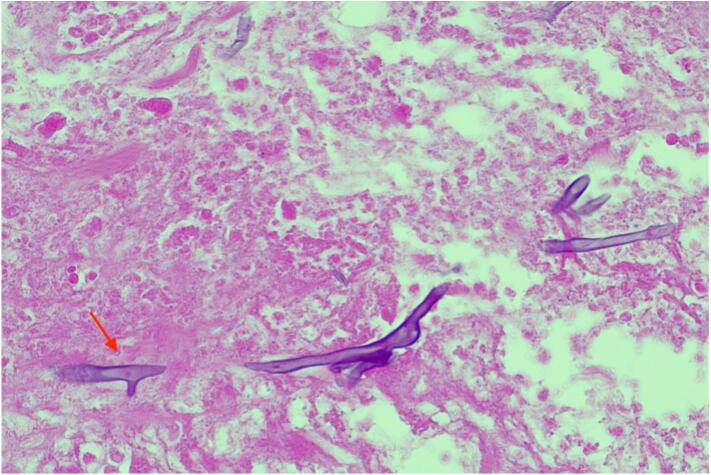
Histological images of 90 degrees branching of hyphae in the wound. 90 degrees branching of hyphae is characteristic of Mucorales order.

**Fig. 3:**
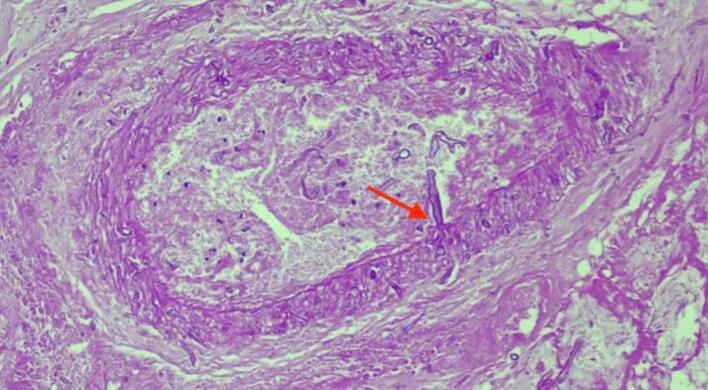
Periodic acid-Schiff (PAS) stained histological images showing invasion of fungal hyphae into the vessel wall (angioinvasion).

**Fig. 4:**
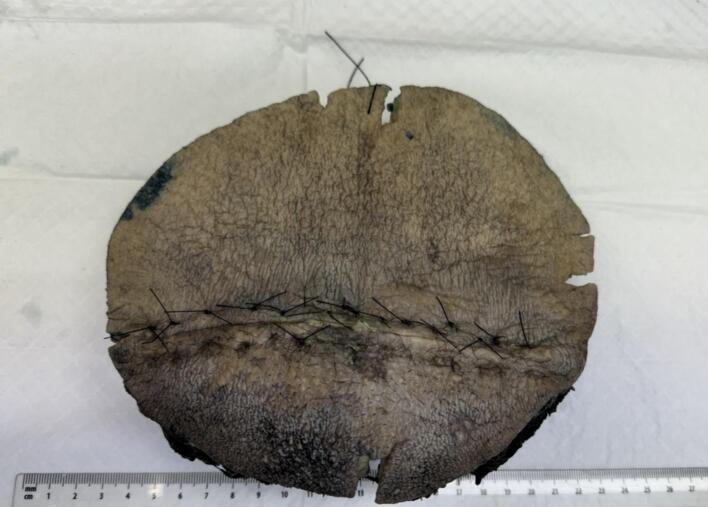
Resection en bloc specimen measuring 194 mm (width) × 169 mm (height) × 41 mm (depth) taken at the pathology laboratory.

On POD two, the patient once again became septic with a rapid spread of cellulitis around the wound. She was admitted to the intensive care unit. On this day, the histology of tissue from her initial debridement identified fungal hyphae with 90-degree branching (Fig. 2) and angioinvasion (Fig. 3), which was pathognomonic of Mucorales order infection, i.e. mucormycosis [8]. Given the above concerns and deteriorating sepsis, the patient underwent a radical surgical debridement. The wound was taken en bloc to ensure adequate margins (Fig. 4). Parts of the anterior rectus sheath and the external oblique aponeurosis were found to be involved in the infection and were sacrificed. Washout with hydrogen peroxide was performed and the wound was packed with iodine-soaked ribbon gauze.

Infectious diseases specialist input was involved throughout the patient's admission. Following the return of culture results on POD two, antifungal monotherapy with intravenous liposomal amphotericin B 450 mg daily was initiated. After one week, a loading dose of oral posaconazole 300 mg twice a day was added and continued with 300 mg daily for combination therapy. This treatment was guided clinically by the patient's response as well as sensitivity results obtained subsequently. Tissue cultures from the earlier debridement were sent to an interstate specialty mycotic laboratory, which found *Saksenaea vasiformis* after a few weeks of analysis. Sensitivities showed a mean inhibitory concentration of amphotericin B and posaconazole were 1 mg/L and 0.06 mg/L respectively.

Piperacillin-tazobactam and lincomycin were ceased on POD 4, following confirmation of mucormycosis in the absence of significant bacterial infection on several swabs, blood cultures and histology, as well as the patient's gradual improvement following initiation of antifungal treatment.

After three weeks, amphotericin B was discontinued due to clinical improvement and concerns with the development of renal toxicity despite aggressive intravenous fluid and electrolyte replacement. The patient had a nadir estimated glomerular filtration rate (eGFR) of 61 mL/min/1.73m^2^ (with a prior baseline >90 mL/min/1.73m^2^). Renal function recovered to baseline gradually following cessation of amphotericin B. The patient continued posaconazole at 400 mg daily for a further three weeks, finishing on POD 39.

Following the initiation of antifungal treatment and radical debridement, the patient began to improve clinically. On POD nine, a vacuum-assisted wound closure (VAC) dressing was applied to the wound. The patient underwent a further six planned operations every five days for reapplication of VAC dressings and minor debridement of any non-viable tissues.

On POD 22, swabs from a routine debridement and reapplication of VAC dressing grew *Pseudomonas aeruginosa* and found scant fungal elements in necrotic regions but not in clear margins of healthy tissue. Piperacillin-tazobactam was reinitiated for a short course of treatment of *Pseudomonas aeruginosa* colonisation for reconstruction planning.

**Fig. 5:**
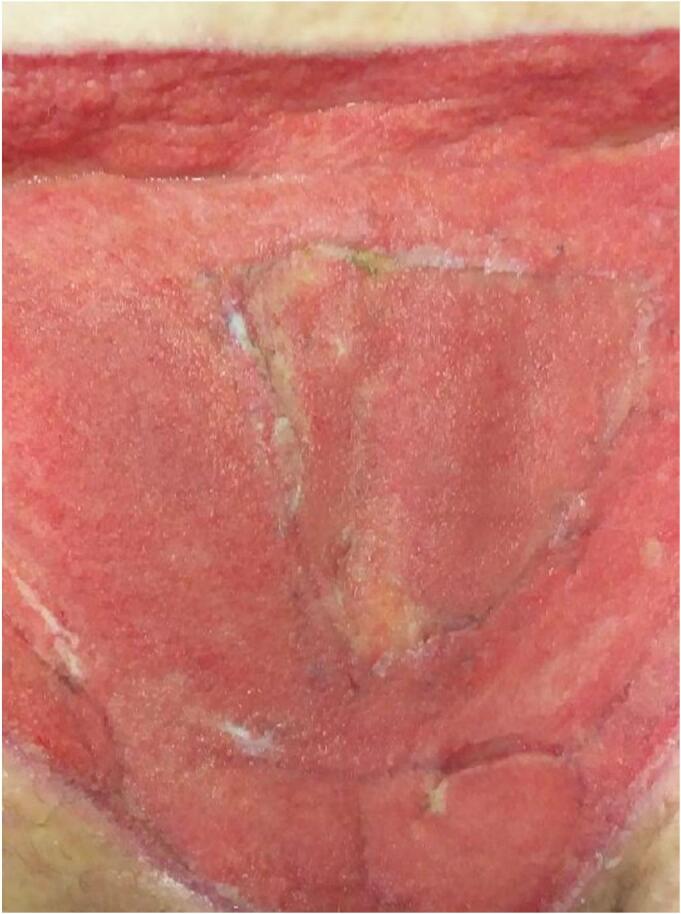
Base of wound two weeks following insertion of Phasix™ Mesh on an onlay method and ongoing VAC dressing treatment.

Reconstruction was planned to repair the en bloc resection of the infected Pfannenstiel wound and the sacrificed anterior rectus sheath and external oblique aponeurosis. On POD 28 the anterior rectus sheath and external oblique aponeurosis were reconstructed using a knitted poly-4-hydroxybutyurate monofilament Phasix™ (C. R. Bard, Inc., Warwick, RI, USA) mesh in an onlay technique and the perimeter was sutured to the remaining fascia (Fig. 5). The patient was eventually discharged on POD 33 with a VAC dressing in situ.

**Fig. 6:**
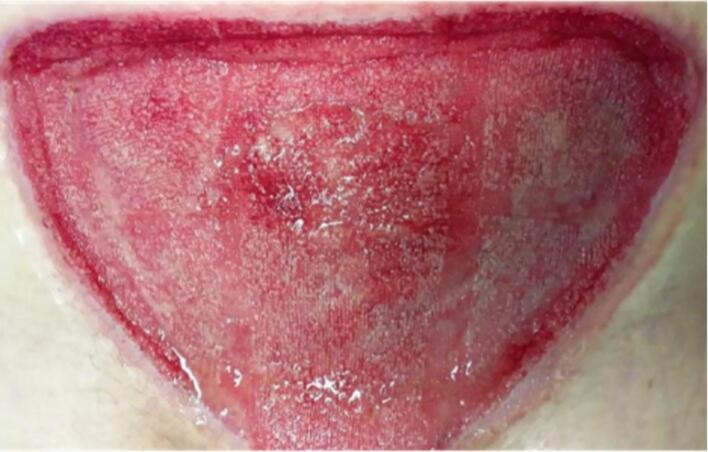
Clinical image of base of wound two months following Phasix™ Mesh insertion with ongoing VAC dressings, just prior to BTM grafting.

**Fig. 7:**
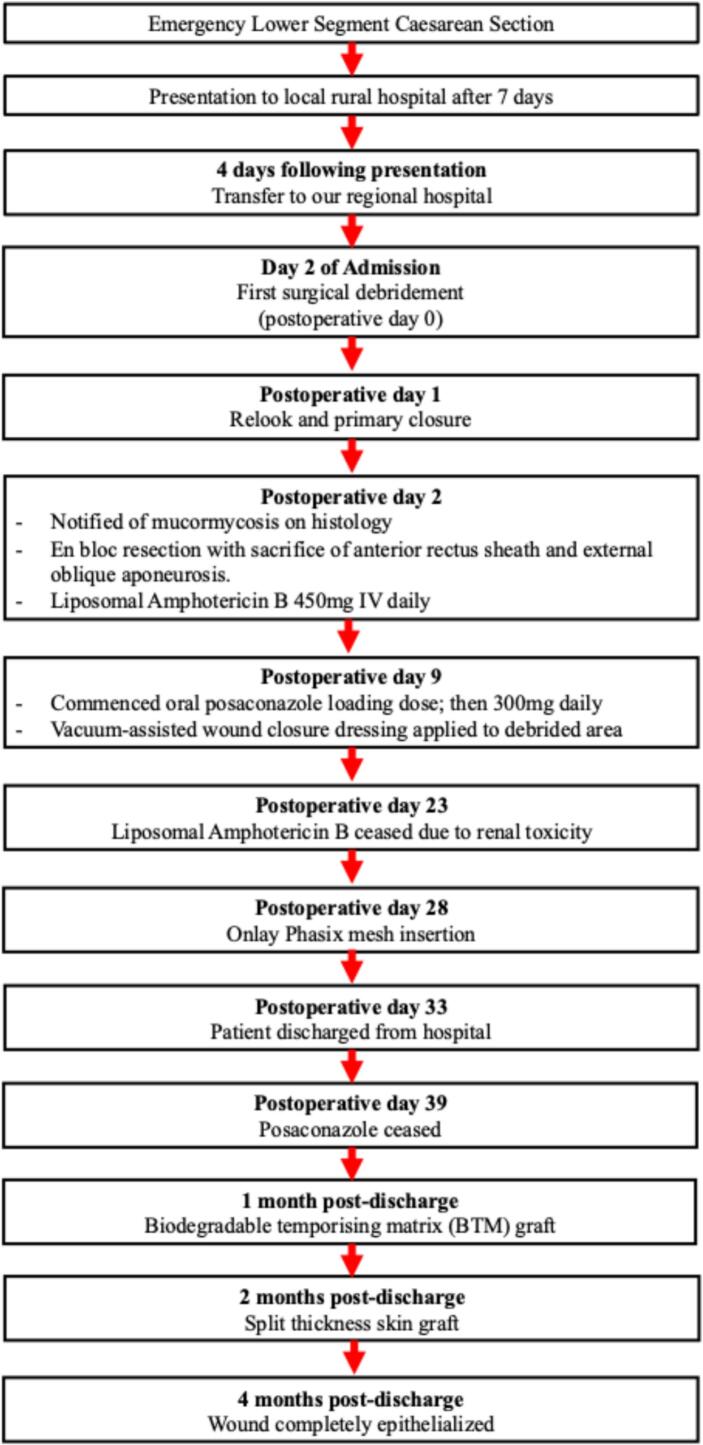
Timeline of major events from presentation to complete wound epithelialization.

One month later, the wound base appeared healthy (Fig. 6) and was grafted with BTM to reconstruct the dermis, followed by split thickness skin grafting four weeks after. Unfortunately, the split-thickness skin graft did not take, and the wound was left to heal by secondary intention with regular acetic-acid-soaked dressings due to *Pseudomonas aeruginosa* colonisation. After a further two months of follow-up in the outpatient setting, the wound eventually epithelialized (Fig. 7). Throughout the reconstruction period, VAC dressings were continuously used.

## 3 Discussion

*Saksenaea vasiformis* most often causes subcutaneous mucormycosis infection [9,10], followed by rhino-orbital cerebral infection, necrotising fasciitis, disseminated disease, pulmonary infection and osteomyelitis [10].

The most common cause of cutaneous infection by *Saksenaea vasiformis* is secondary to trauma [11], following introduction of fungal spores to the disrupted skin barrier [12]. *Saksenaea vasiformis* infection has also occurred following lacerations, health-care related wounds, burns, abrasions, animal/insect bites and tattooing [10,12]. More than 80 % of infections caused by *Saksenaea vasiformis* occur in healthy non-immunocompromised patients [11]. In this case, the patient likely has elements of immune modulation from the recent pregnancy, compounded by her post-partum haemorrhage. The patient was plausibly subjected to an immunocompromised state in this setting, which would increase the risk for atypical infections such as mucormycosis.

Similar to other causes of mucormycosis by the Mucorales order, histological findings of angioinvasion by hyphae and tissue necrosis are characteristic of *Saksaenaea vasiformis* infection [8]. Culture of this fungus and identification by this microscopic morphology may take several weeks due to difficulty in achieving sporulation [8]. Given the technical challenges in identifying *Saksaenaea vasiformis*, diagnosis of the condition is difficult and may be underrepresented in literature. As evidenced in this case, histological diagnosis was ultimately required as other methods of investigation, including wound swabs and blood cultures, remained negative despite clinical evidence of sepsis. Hence, in the presence of progressive and clinically obvious soft tissue infection and sepsis despite treatment with broad-spectrum antibiotic therapy, differential diagnoses of infection from atypical organisms such as *Saksaenaea vasiformis* should be considered.

Treatment for mucormycosis, including *Saksaenaea vasiformis*, typically includes a first-line agent of intravenous liposomal amphotericin B [3] alone or in combination with oral posaconazole therapy [10]. As a single agent or as part of a combination regimen, posaconazole therapy may have an added survival benefit [10]. The European Confederation of Medical Mycology and the Mycosis Study Group Education and Research Consortium guidelines strongly recommend aggressive debridement with liposomal amphotericin B as a first-line agent. A moderate recommendation is made for consideration of posaconazole either as a first-line agent when amphotericin B toxicity occurs or as an oral long-term maintenance option [13]. Survival without antifungal treatment is markedly poor [3,10]. Most patients require aggressive surgical debridement of their mucormycosis infection [3,10]. With little evidence in the literature, paired with limited clinical information during the admission, we erred on the side of caution and performed a radical surgical debridement of the mucormycosis infection whilst treating with both amphotericin B and posaconazole.

By sacrificing the anterior rectus sheath and external oblique aponeurosis, the anterior abdominal wall is weakened with possible sequelae of hernias. Therefore, after ensuring resolution of the mucormycosis infection, we opted for an onlay mesh repair with a Phasix™ mesh, which is made from a biologically derived, fully resorbable material. Given the resorbable nature of Phasix™ mesh, we thought it was appropriate for use in this infected setting, with *Pseudomonas aeruginosa* growth on wound swabs and histological evidence of scant fungal elements within necrotic tissue from debridement six days prior to mesh insertion. Studies have shown that the use of Phasix™ mesh in a contaminated field is safe with similar long-term outcomes [14]. Likewise, BTM is also clinically shown to be robust in the presence of infection [15]. Alternative options such as locoregional flaps may be considered when there is a need for complex reconstruction of musculofascial layers of the abdominal wall [16].

Overall, considering the size of the debrided area and simple anterior fascial defect, we felt that a staged reconstruction with mesh, BTM and split thickness skin grafting over any type of flap repair would be more beneficial and less harmful for the patient following the reconstructive ladder principle. This is with an aim to heal the defect as quickly as possible, minimise invasiveness, reduce further infection and decrease metabolic burden to the patient [17].

## 4 Conclusion

This case highlights the exceptionally rare and difficult diagnosis of mucormycosis of a lower segment caesarean section wound, ultimately requiring histological diagnostic confirmation. Radical surgical debridement with antifungal treatment with liposomal amphotericin B with posaconazole was essential for treatment. This case also describes the reconstruction of a large abdominal defect and the sacrificed anterior rectus sheath and external oblique aponeurosis in a potentially infected setting in this rare condition.

## Author contribution

Steven NGUYEN: study concept or design, data collection, data interpretation, writing the paper, revision and editing of paper.

Hao Han TAN: study concept or design, data collection, data interpretation, writing the paper, revision and editing of paper.

Damian FRY: data interpretation, supervision, revision and editing of paper.

## Patient consent for publication

Written informed consent was obtained from the patient for publication of this case report and accompanying images. A copy of the written consent is available for review by the Editor-in-Chief of this journal on request.

## Ethical approval

Exemption given by Darling Downs Health Human Research Ethics Committee.

## Guarantor

Steven Nguyen.

## Research registration number

Not applicable.

## Funding

This case report did not receive any specific grant from funding agencies in the public, commercial, or not-for-profit sectors.

## Conflict of interest statement

None declared.
